# Understanding experiences of neglected tropical diseases of the skin: a mixed-methods study to inform intervention development in Ethiopia

**DOI:** 10.1136/bmjgh-2024-016650

**Published:** 2025-02-05

**Authors:** Mirgissa Kaba, Yohannes Hailemichael, Abebaw Yeshambel Alemu, Teklu Cherkose, Getachew Kebebew, Fikregabrail Aberra Kassa, Galana Mamo Ayana, Tedros Nigusse, Kibur Engdawork, Zenebu Begna, Abay Waday, Tara B Mtuy, Saba Lambert, Katherine Elizabeth Halliday, Maria Zuurmond, Rachel L Pullan, Stephen L Walker, Catherine Pitt, Endalamaw Gadisa, Michael Marks, Jennifer Palmer, SHARP Collaboration, Yaw Amoako

**Affiliations:** 1 School of Public Health, Addis Ababa University, Addis Ababa, Ethiopia; 2 Armauer Hansen Research Institute, Addis Ababa, Ethiopia; 3 Department of Epidemiology and Biostatistics, University of Gondar, Gondar, Ethiopia; 4 Department of Sociology, Debre Markos University, Debre Markos, Amhara, Ethiopia; 5 Department of Sociology, Addis Ababa University, Addis Ababa, Ethiopia; 6 Department of Public Health, Ambo University, Ambo, Ethiopia; 7 School of Population Health, Curtin University, Perth, Western Australia, Australia; 8 Department of Clinical Research, London School of Hygiene & Tropical Medicine, London, UK; 9 Department of Disease Control, London School of Hygiene & Tropical Medicine, London, UK; 10 Department of Non-communicable Disease Epidemiology, London School of Hygiene & Tropical Medicine, London, UK; 11 Department of Global Health and Development, London School of Hygiene & Tropical Medicine, London, UK; 12 University College London, London, UK

**Keywords:** Leprosy, Cutaneous leishmaniasis, Health services research, Qualitative study, Health economics

## Abstract

**Background:**

The WHO and Ethiopia’s Ministry of Health have developed strategies to expand and integrate services for co-endemic neglected tropical diseases (NTDs) which manifest in the skin. To inform these strategies, we aimed to understand the social, economic and health system context of skin NTD care in Kalu woreda, Amhara region, Ethiopia, where cutaneous leishmaniasis (CL) and leprosy are endemic.

**Methods:**

Between October 2020 and May 2022, we surveyed and reviewed records of 41 primary healthcare facilities and explored common disease experiences in focus group discussions (n=40) and interviews with people affected by leprosy (n=37) and CL (n=33), health workers (n=23), kebele authorities and opinion leaders (n=33) and traditional healers (n=7). Opportunities for integrated skin NTD service provision were explored through policy document review, interviews with health officials (n=25), and stakeholder meetings.

**Results:**

Availability of diagnostic supplies and health worker competence to provide skin care was very limited across primary healthcare facilities, particularly for CL. People with leprosy commonly sought care from healthcare facilities, while people with CL administered self-care or sought help from traditional healers. Travel and costs of care at specialised facilities outside the district inhibited timely care-seeking for both diseases. Transmission discourses shaped different understandings of who was affected by leprosy and CL and expectations of behaviour during and after treatment. Many policy actors felt that existing supply chain interventions, decentralised treatment approaches and community engagement initiatives for leprosy could also benefit CL, but others also warned against increasing care-seeking unless CL treatment could be provided on a scale commensurate with the large burden they perceived.

**Conclusion:**

Our findings demonstrate significant gaps in the provision of care for skin NTDs within primary healthcare, very different health-seeking patterns for leprosy and CL, and a need to develop new models of care, especially for CL.

**WHAT IS ALREADY KNOWN ON THIS TOPIC**

- In Ethiopia, leprosy services have been fully integrated into general health services and could serve as a model for integrating services for other neglected tropical diseases of the skin (skin NTDs) such as cutaneous leishmaniasis.
- Evidence is needed on local healthcare delivery contexts to enable integration of each skin NTD.

**WHAT THIS STUDY ADDS**

- In a rural district of Amhara region, we found major gaps in primary healthcare provision for both diseases, very different health-seeking patterns for leprosy and cutaneous leishmaniasis, and policy actor concerns about the feasibility of expanding treatment services for cutaneous leishmaniasis outside of dermatology centres.
- The challenges outlined contribute to low existing provision and use of cutaneous leishmaniasis services in the district and will need to be addressed in future integration plans.

**HOW THIS STUDY MIGHT AFFECT RESEARCH, PRACTICE OR POLICY**

- Our findings have informed initial piloting of new community engagement approaches and innovative models of care for CL within primary healthcare in Ethiopia and should encourage skin NTD integration strategies in all countries to be tailored to local contexts.

## Introduction

 Many neglected tropical diseases (NTDs) manifest primarily in the skin and are referred to as ‘skin NTDs’.^1^ Disability and stigma associated with skin NTDs contribute to social exclusion and economic hardship.^1 2^ Several skin NTDs, including leprosy and cutaneous leishmaniasis (CL), are endemic in Ethiopia. With 2966 leprosy cases reported in 2022, a considerable burden of disability due to late diagnosis persists and cases in children indicate recent or ongoing transmission.^3^ More than 20 000 CL cases are estimated to occur annually, although the precise burden is difficult to ascertain because of limited care-seeking through formal health services and limited diagnostic capacity.^4^,^6^

The WHO and Ethiopia’s Federal Ministry of Health (FMoH) have developed policies to integrate prevention, diagnosis, treatment and care of co-endemic skin NTDs,^4^ but evidence is required to inform their design and implementation. Ethiopian leprosy services have been fully integrated into general health services. Although some aspects are suboptimal, leprosy services might serve as a model for some aspects of integration for other skin NTDs including CL.^7^,^10^ In contrast to leprosy, CL is currently managed at a few specialised treatment centres, which treat less than 5% of CL cases annually.^4 11 12^ Ethiopia has adopted an ambitious objective to detect 85% of CL cases and treat 95% of those detected by 2030 through rapidly strengthening services. Plans include expanding the number of specialist facilities providing diagnostic services from 25 to 170, expanding the number providing treatment from 14 to 30 and building public awareness of the increased capacity. This ambitious programme requires investment in human, diagnostic and therapeutic resources, and in enhancing service accessibility in areas of need.^4^

Integrated approaches are complex and require tailoring to local contexts, including disease distributions, existing healthcare provision, the ways individuals and communities experience diseases and care services, and the ways planners imagine care could be organised.^13^ To inform the design of integrated approaches to skin NTDs, we undertook formative research on the social, economic and health system context of skin NTD care in Ethiopia’s Amhara region through the Skin Health Africa Research Programme (SHARP).

## Methods

### Study setting

**Figure 1:**
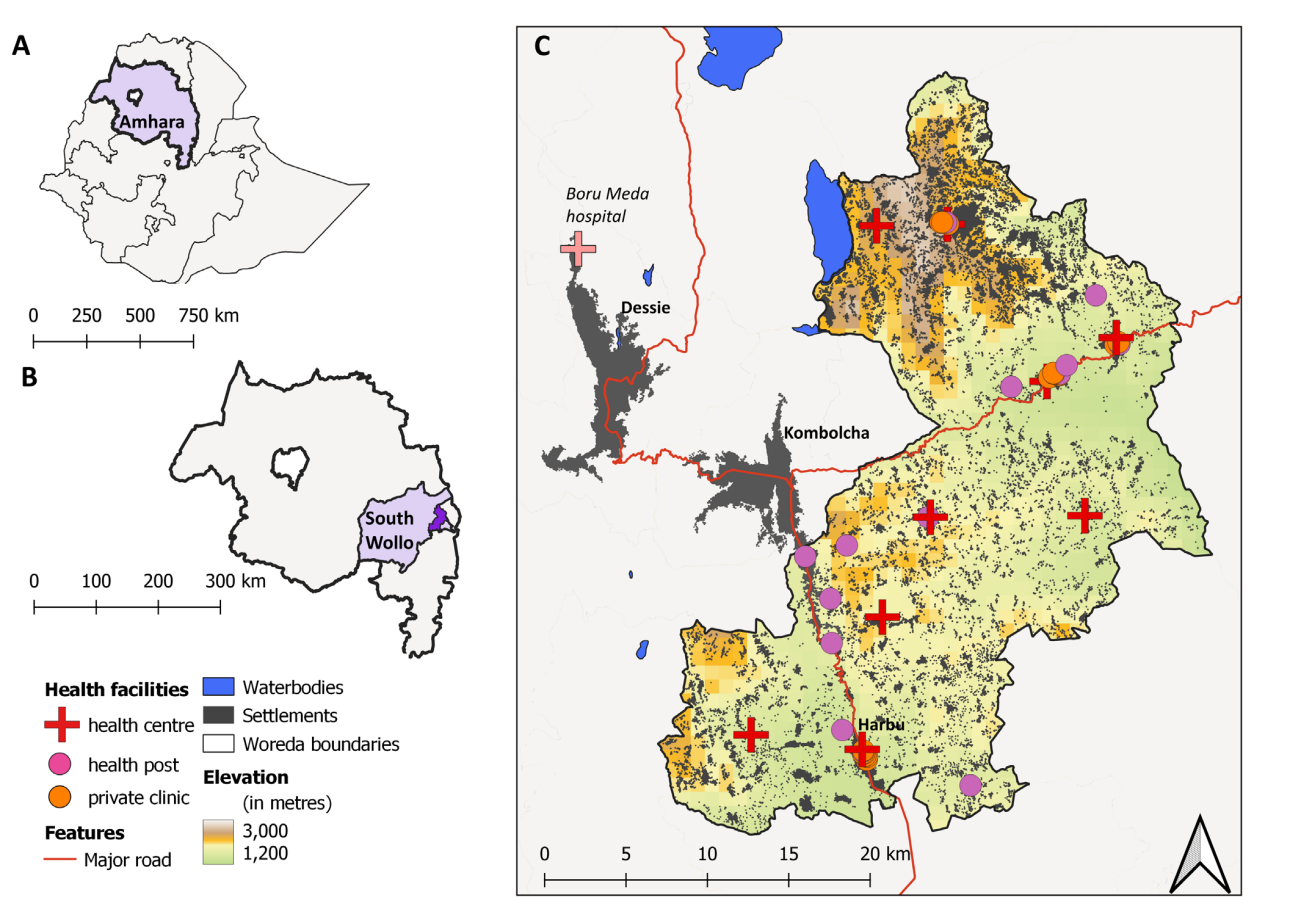
Map of the study area showing location of (**A**) Amhara region within Ethiopia and (**B**) Kalu woreda within the South Wollo Zone, Amhara; (**C**) location of health facilities visited through the study within Kalu. Background shading reflects population density (in grey) alongside elevation, with the majority of the woreda lying between 1650 m and 2560 m (CL in Ethiopia is reported to occur at this elevation).^38^ Basemap was generated in qGIS using district boundary files provided by GADM (https://gadm.org/) and population and elevation data provided by GRID3 (Ethiopia Settlement Extents V.2.0: https://data.grid3.org/datasets/GRID3::grid3-eth-settlement-extents-v2-0/explore) and CGIAR-STRM (https://srtm.csi.cgiar.org/). These data sources are all provided free of charge, without restriction of use (CC BY 4.0).

The study was conducted in Kalu woreda (district) (population 237 319 in 2020) in the South Wollo zone of Amhara region. Kalu consists of 40 ‘kebeles’ (small administrative units), of which 35 are rural and 5 urban; the kebeles are grouped into nine clusters to organise provision of health and other services.^14^ Reports from neighbouring referral facilities suggested the presence of CL and leprosy in Kalu, but neither the FMoH nor development partners had implemented any interventions targeting skin NTDs. Kalu includes areas of highland and lowland terrain (figure 1). The population encompasses several ethnic groups, and most residents are Muslim. Subsistence agriculture and livestock rearing are the major economic activities.

Primary care is delivered through 9 government-run health centres (one per cluster) with limited inpatient capacity and 35 health posts staffed by health extension workers, which provide essential health promotion and prevention services but limited treatment services. Several private-for-profit clinics serve the larger population centres. There is no hospital in the woreda. The nearest hospital is in Dessie while the closest dermatology referral centre is Boru Meda general hospital; both are approximately 50 km from Harbu, the administrative centre of Kalu.

The data collection period was affected by COVID-19 restrictions and armed conflict. Kalu woreda was not directly affected by the conflict, but curfews and checkpoints affected transportation. Research team members avoided discussing topics that could be interpreted as political, including asking about how the conflict might affect service provision and care-seeking.

### Study design

We used a concurrent mixed-methods design described below. In October and November 2020, prior to data collection, we consulted officials from the FMoH and regional, zonal and woreda health authorities. In each cluster, meetings were held with kebele administrative authorities, community opinion leaders (religious leaders, youth leaders and women’s health development army leaders) and health extension workers to discuss the study and seek permission to work. Data were collected from March to October 2021 in three parallel streams covering health service readiness, care-seeking practices and the policy landscape with specific methods described below. Findings from all streams were discussed at a series of research team meetings to identify commonalities and points of divergence in themes arising from different study phases, methods and theoretical orientations. In May 2022, further meetings were held with national, zonal, woreda and community-level stakeholders to reflect on the findings and discuss the design of future interventions.

#### Health service readiness survey and records review

We conducted health facility assessments from July to October 2021 to assess both general and NTD-specific service ‘availability’ (whether facilities offer a variety of preventive and curative health services) and ‘readiness’ (whether facilities have the items and skills required to deliver that service at the time of the site visit). The assessment was carried out in all 9 FMoH health centres in the woreda, as well as 14 of 35 health posts and 18 private-for-profit clinics based in the four larger towns. The study team were not able to visit the remaining 21 health posts as they were inaccessible due to rains. During these assessments, we interviewed the facility lead, the person in charge of disease-specific services, and/or an alternative, suitably informed staff member. Data were collected using Open Data Kit,^15^ encrypted and stored on password-protected servers.

Assessments were based on standardised WHO indicators describing health infrastructure and health workforce (in both public and private facilities), basic amenities, equipment, infection prevention and control activities, diagnostics and availability of essential medicines (in public facilities only).^16^ We developed specific modules for CL and leprosy, following the model of the WHO Direct Inspection Protocol for lymphatic filariasis^15^; domains encompassed availability of trained staff and education materials, as well as diagnostics, treatment and case management services. Each domain was defined based on the presence or absence of the recommended standard of care in Ethiopia. CL- and leprosy-specific modules were applied in both public and private facilities.

We reviewed routine health surveillance records at seven of the nine health centres for the 12-month period 1 August 2020 to 31 July 2021; the remaining two health centres were not accessible at the time of the records review. Health posts and private facilities were excluded because of the observed low use of health data reporting systems. Health facility staff were initially consulted to identify all methods for recording leprosy and CL data at each facility. We reviewed all disease-specific case reporting forms, where available, and extracted case-level data. Additionally, general outpatient department registers and aggregated reports (ie, monthly and annual totals) were reviewed, with all common skin disease and skin NTD cases documented.

#### Skin NTD care-seeking practices

To describe popular discourses around CL and leprosy, we held 40 focus group discussions (FGDs) with community members from villages across Kalu woreda (4–5 per cluster) and interviews with 33 kebele authorities and community opinion leaders (3–4/cluster), 23 health workers (one per facility participating in the readiness survey) and 7 traditional healers. We interviewed 37 people confirmed to have leprosy and currently under the care of health centres in Kalu (receiving multidrug therapy (MDT) or post-MDT management). As no CL diagnoses were identified in the health centres record review, we interviewed 33 people affected by CL identified by key informants (health facility staff, community leaders and other affected people). None of these individuals were clinically assessed; around one-third had active skin lesions. Interviewees presumed to have CL were informed about services available at Boru Meda Hospital.

Interviews with leprosy- and CL-affected people explored their experiences of disease recognition, beliefs about causation and transmission, treatment decisions and experiences of living with the diseases, including the household economic burden and experienced stigma. For affected children, parents or guardians were interviewed. All research activities took place in Amharic. Additional sampling and recruitment information is available in online supplemental file 1.

#### Skin NTD policy landscape

To understand the challenges and opportunities related to integrated skin NTD service provision, we reviewed 15 policy documents, including health sector development strategies and NTD plans (for a list of documents, see online supplemental file 2); interviewed 25 health officials working at national, regional, zonal and woreda levels (for sampling details, see online supplemental file 1) and took notes during stakeholder meetings.

Additional detail on dynamics between the study team and participants and on qualitative methods is available in our Consolidated Criteria for Reporting Qualitative Research checklist^17^ (online supplemental file 3).

### Data management and analysis

#### Quantitative data analysis

The service availability and readiness assessment survey data were summarised using RStudio (V.4.0.4). Outcomes were coded as binary indicators describing the characteristics of health facilities and service provision for basic health services and skin NTD services. Total scores for each domain were calculated as the mean score of these binary indicators and standardised as a score out of 100 for that domain. We calculated the mean of each domain score and expressed the general and skin NTD service readiness indices as scores out of 100 for each health facility. Finally, overall mean scores for the woreda were calculated as a mean of facility averages. We tabulated cases reported across case reporting forms and facility registers. Results were summarised by facility and for the woreda, with frequencies and percentages. In cases where there were discrepancies between recording types, the greater case load was used.

#### Qualitative data analysis

Qualitative data were transcribed verbatim in Amharic, translated to English and coded thematically with MAXQDA software.^18^ A coding framework was developed according to preagreed themes (online supplemental file 4). Data were first coded to allocate material across themes and then distributed to a thematic lead researcher who was responsible for further coding, synthesis and interpretation through discussion with the wider team. For the care-seeking stream, only a proportion (around half) of transcripts from each participant category (online supplemental file 1) were fully analysed in time to inform intervention development, and this analysis is presented here. The samples analysed were chosen to maintain a similar demographic balance as the original sample and to be large enough to reach theoretical saturation on the main themes reported here. All interviews with healers were analysed because of the small sample size achieved. Additional manuscripts drawing on this data have been prepared to look in more detail at the traditional treatment and stigma experiences of people with CL (in preparation) and the role of economic factors constituting the household burden of skin NTDs across Ethiopia and Ghana.^19^

### Patient and public involvement

Leprosy- and CL-affected people, communities and their representatives were consulted on the research design, research questions and how best to disseminate findings. The selection of study sites and inclusion of participants was led by local government staff and community leaders. Findings were discussed in meetings that included leprosy- and CL-affected people.

## Findings

### Health service availability and readiness

#### Basic health service readiness

Public health facility density was 94% of the WHO-recommended threshold at 1.85 facilities per 10 000 population, although this density dropped to 0.5 per 10 000 when considering only health centres.^20^ The number of clinically trained health workers within health centres and private facilities (13.3 per 10 000 population) was 55% of the WHO-recommended minimum. Only one government health centre and three private facilities in the woreda employed a doctor.

Overall service readiness (a composite score comprising basic amenities, diagnostics, equipment, essential medicines and infection prevention and control) across all woreda health centres was 83, that is, a mean of 83% of the indicator criteria were met. Service readiness across health posts visited was far lower at 39 (table 1). Differences between health centres and health posts were greatest for diagnostics and equipment and least pronounced for infection prevention and control measures. Routine diagnostic capacity in health centres was high: all had HIV, syphilis and malaria rapid diagnostic tests (RDTs) in stock. Health posts surveyed stocked only malaria RDTs. No facilities had a dermatoscope. Essential medicines were generally well stocked at health centres, while health posts stocked a more limited range. Only 6 of the 14 posts visited stocked wound dressings.

**Table 1:** Domain scores and overall composite index scores for general readiness and leprosy- and cutaneous leishmaniasis (CL)-specific readiness for health centres, health posts and private clinics in Kalu woreda

Domain	Indicators and description	Health centre (n=9)	Health post (n=14)	Private clinic (n=18)
General readiness index				
Basic amenities	Communication, computer, emergency transport, improved water, power, private consultation, sanitation facilities	70	43	Not assessed
Equipment	Blood pressure, stethoscope, tape, thermometer, torch	91	33	Not assessed
Infection prevention and control (IPC)	Clean gloves, disinfectant, hand washing facility, IPC guidelines, safe waste disposal, sharps bin, syringe	78	57	Not assessed
Diagnostics	Glucose dipstick, HIV, malaria, pregnancy stick, protein dipstick, syphilis	91	19	Not assessed
Essential medicines	Analgesics, anthelmintics, antimalarials, diabetes, family planning, first aid kit, heart disease, HIV-ARVs, hypertension, injectable antibiotics, oral antibiotics, ORS, vaccines, wound dressing	88	44	Not assessed
Overall composite index score		83	39	n/a
Leprosy readiness index				
Training	Clinical staff based at facility trained in diagnosis and treatment in the last 2 years	39	0	10
Education and guidelines	Guideline covering diagnosis, treatment and/or management available for reference	11	0	0
Diagnostics	Clinical diagnosis offered	100	0	35
Treatment	Multi-drug therapy offered and in stock	83	0	0
Patient tracking	New suspected and confirmed cases are recorded at facility.	100	14	35
Community management	Active case-finding, contact-tracing	0	86	0
Staff knowledge	Knowledge of cardinal signs of PB and MB, disability identification, treatment	56	0	1
Overall composite index score		56	14	12
CL readiness index				
Training	Clinical staff based at facility trained in diagnosis and treatment in the last 2 years	0	0	0
Diagnostics	Clinical diagnosis offered, laboratory confirmation performed, appropriate referrals made	19	31	20
Treatment	Wound dressings, CL treatments (topical, intravenous, cryotherapy or intralesional)	50	12	0
Overall composite index score		23	16	7

Scores are standardised, to lie between 0 (no tracer indicators present in any facility) and 100 (all tracer indicators present in all facilities).

ARVAnti-retroviralMBMultibacillaryORSOral rehydration solutionPBPaucibacillary

#### Health service readiness to provide care for leprosy and CL

Compared with overall service readiness, leprosy- and CL-specific readiness scores were markedly lower (table 1, onlinesupplemental files 5  6). For both conditions, readiness scores were lowest at private facilities (where, on average, fewer than 12% of tracer indicators were met), and only a few points higher at health posts. Readiness at health centres was higher for leprosy (with an overall score of 56) than for CL (overall score 23).

For leprosy, respondents at all nine health facilities stated they were able to offer clinical diagnosis, and seven of nine stocked MDT. However, only one health centre had guidelines available for staff reference, and only four had staff who had received training in diagnosis or management of leprosy in the past 2 years. Of 18 private facilities, respondents at 7 stated they were able to offer clinical diagnosis of leprosy, but no staff surveyed from these facilities were able to recognise signs of leprosy or recall the correct treatment. Respondents at health posts stated they were not able (and it is not part of their responsibilities) to offer diagnosis or treatment for leprosy; however, health extension workers (who staff health posts) were involved in community leprosy management. Of 14 health posts surveyed, respondents at 13 reported that they conducted active case-finding and 11 contact-tracing.

For CL, diagnostic and treatment capacity was considerably lower. Respondents at only two health centres stated they could offer clinical diagnosis, and three that they made appropriate onward referrals. Some health posts and private clinics also reported capacity for clinical diagnosis and referral. No health personnel in surveyed facilities had received any training on diagnosis or management of CL, and all reported that they were not expected to provide treatment at their facilities.

#### Health service data on skin NTD prevalence

All health centres visited recorded potential and confirmed leprosy cases in a dedicated register and reported to the woreda health office. From August 2020 to July 2021, six health centres recorded 30 confirmed leprosy cases (new case detection rate: 1.3 per 10 000 people per year; 1–10 cases per centre). Over the same period, health centres recorded no CL cases. No facilities surveyed had case-based reporting forms for CL available.

### Discourses about disease recognition, causation, and care in Kalu

In Kalu, the symbolism associated with both leprosy and CL was emotive but differed. Leprosy was linked to swampy lowlands, *jinns* (evil spirits), soil, meat and unhygienic living conditions, while CL was associated with mountains, bats, the moon, ventilated housing, and sexual intercourse.

#### Leprosy

The Amharic for leprosy, *Siga Dewye/Yesiga Dawe,* literally meaning ‘disease affecting skin and muscle’, was well known, as was the Arabic translation, *Juzan,* among Muslim community members. The commonly recognised early indication of leprosy was skin pallor. Other features included itchy, painful or swollen lesions. Loss of function and changes to the shapes of hands and feet were recognised as advanced signs. Interviewees with leprosy often discussed numbness and body parts becoming ‘tired and weak’.

People described multiple causes or risk factors for developing leprosy, including dirty and stagnant water, where *jinns* are believed to exist; walking barefoot in swampy areas; curses and inherited traits. Most respondents reported that they believed men, the elderly and those living in rural, impoverished conditions to be at greater risk of acquiring leprosy.

Leprosy was commonly conceptualised as communicable. Some people in FGDs stated that transmission is interrupted once treatment starts. Contact with soil, however, was reported by some to worsen leprosy:

A person with leprosy should avoid contact of lesions with the soil since contact with soil aggravates the pain. (KII-23, male community opinion leader, Harbu)

Most leprosy-affected participants considered health facilities the best source of care for leprosy. However, some individuals affected by leprosy described combining allopathic and traditional care. Only one traditional healer reported offering a treatment for leprosy (a purgative plus dietary restrictions), while the remainder discussed the effectiveness of hospital-based treatment:

As knowledge grows and spreads, we are now seeing this disease disappear … it’s vanishing because of the treatment. (KII-9, traditional healer, Degan)

#### Cutaneous leishmaniasis

CL was known as ‘*kunchir*’ or ‘*konchir*’ and recognised as a common disease in Ketetya and Ardibo, highland clusters of Kalu. CL was well known in other parts of the woreda, but reportedly did not commonly affect residents. CL was said to be identifiable through lesions with darkening of the skin, inflammation, redness and moist wounds. CL was recognised to cause scars or in severe cases deformity of the nose, other facial structures and limbs. One CL-affected individual described his symptoms:

… it [CL] is red without hurting but it expands … this is the character of Kunchir. (IDI-23, man affected by CL, Ketetya)

Participants believed that living in highland areas and young age were risk factors for developing CL. CL was attributed to multiple causes including the wrath of God, insect bites (mosquitoes and tsetse flies were mentioned, but not sandflies) and germs carried by hyraxes. Contact with bat body fluids was the predominant hypothesis of causation among community members, as described in this person’s illness narrative:

A bat entered my home … and flew over me, dropping its urine on me. I didn't feel anything at the time, so I simply cleaned the urine … but after a week, I saw the signs [of CL]. (IDI-23, man affected by CL, Ketetya)

The most common measures taken to prevent CL were directed against bats (killing them, modifying houses to prevent bat entry and sleeping covered at night). Adolescents were said to be more exposed to bats because they have hot bodies and sleep naked. Most people believed CL was not communicable, although a minority believed it was possible for CL to be acquired via direct contact with the blood or sweat of an affected person with active lesions.

Among 18 people interviewed with CL, only one reported to have received care from a health facility (Boru Meda Hospital). People mainly self-administered traditional treatments or sought care from traditional practitioners. Traditional healers spoke about learning from Islamic religious scholars and other practitioners with knowledge of medicinal plants. Plant materials such as garlic, leaves of the tree *Croton macrostachyus*, khat and the sap of *Euphorbia abyssinica [kulkuwal],* were used to treat CL, with more severe wounds tending to be treated with more caustic plant materials. One CL-affected individual described applying soil from a sacred place believed to have healing qualities to his lesions; another described grinding up and applying the desiccated body of a bat.

The use and effectiveness of heat to cauterise lesions was commonly mentioned:

Someone who had ‘kunchir’ told me that he was cured with the application of heat. I did the same. I heated a sickle and when it got very hot, I put it on the lesion. The burned area dried and slowly the ‘kunchir’ disappeared leaving a scar. (IDI-21, man affected by CL, Ketetya)

A majority of participants believed lesions were more bothersome during the full moon and that the effectiveness of traditional CL treatment sometimes depended on affected people abstaining from sexual intercourse. Many individuals with CL described confining themselves to their homes and even avoiding visitors to prevent any ‘shadow’ (*tila*) being cast on them which could hinder healing; such shadows could be cast inadvertently by others who were unclean, for example, because they had had sexual intercourse the same day or were menstruating.

### Stigma associated with CL and leprosy

Participants perceived leprosy and CL as disfiguring diseases, which led affected individuals to experience social and internalised stigma and hampered social interaction:

I don’t have a house. I used to live with people whom I worked for until I got this disease [leprosy]. People rejected me when I got this disease. [After that,] my daughter asked our relatives to build this house here [a small, isolated cottage in the periphery of Harbu town]. When I was healthy, everyone loved me. Now, everybody hates me. (IDI-86, woman affected by leprosy, Harbu)

Individuals with scars and especially active CL lesions or anatomical changes from leprosy experienced internalised stigma. Affected people described feeling shame or embarrassment, and commonly reported reduced feelings of self-worth and fear of physical impairments being seen, which resulted in self-exclusion from social activities.

Among persons affected by leprosy and CL, concealment was a common coping strategy. Individuals avoided conversations about their condition and covered the affected body part. A woman with leprosy explained:

Normally, I don't talk about this [leprosy] to anyone including my family. I just keep it to myself and conceal it. (IDI-1, woman affected by leprosy, Gerba)

### Economic burden of skin NTDs

Participants described care-seeking for CL and leprosy as imposing a substantial economic burden on affected individuals and their household. This burden led to delays in seeking diagnosis and treatment at formal facilities and reduced adherence to prescribed medicines. Out-of-pocket costs for transport and medicines were particularly burdensome for individuals with CL seeking care from a hospital outside the woreda. One discussion participant with CL described his stay at Boru Meda Hospital for treatment lasting 3–4 weeks and costing 3000 Birr ($56) ‘just for medical treatment’ (FGD-21, male, Ketetya). Both sodium stibogluconate for CL and MDT for leprosy are provided free of charge, but households had to pay for other supplies to administer care, medicines required for leprosy-associated complications, investigations and in-patient stays, as well as food and accommodation en route to and at the health facility:

Our community lacks money to go to Boru Meda Hospital. Some who have the money go and others don’t. There are some who can’t afford the transportation cost and are forced to just live with the pain. (FGD-2, male, Gerba)

In contrast, CL treatment provided by traditional healers was described as more accessible and affordable and traditional healers themselves often conceptualised flexible pricing (based on ability to pay) as part of their religious commitment to charity:

There is no other treatment in our locality. I heard there is treatment in Dessie [… but] I don’t even have an asset to sell. So, looking for local healers is the only option … they understand your [financial] problem. (IDI-30, woman with CL, Ketetya)

[the imams] pray for me … as I do charity work [give free treatment] for poor and helpless people, which is important for my eternal soul to have rest with peace after death. (IDI- 45, traditional healer, Ketetya)

CL and leprosy were frequently described as imposing substantial opportunity costs. Affected individuals and their caregivers described missing paid and/or unpaid work, most commonly farming, as well as school, both when seeking care and over the course of their illness. Men were expected to accompany women and children to seek care, especially when travelling beyond the local area. Time away from productive activities was also a concern for people in self-enforced isolation during CL treatment and for people with leprosy who feared contact with soil during their usual farming activities.

A variety of community-level risk-pooling arrangements offered some support. Ethiopia’s community-based health insurance was described alongside several traditional risk-pooling mechanisms, notably *debo* (the donation of farm labour by community members to those in need)*, eder* (a traditional form of insurance) and *equp* (a traditional social investment arrangement), as well as informal donations from community members to affected households:

If a person is sick, whether it is a skin disease or other things that make a person bed-ridden, and if that person has work on the farm, the work is covered by the communal labour. We call it ‘debo.’ (FGD-9, male community member, Ressa)

Community leaders and members appeared more confident in the adequacy of these systems than those from affected households. Affected individuals and their caregivers described a lack of community support and reported resorting to household-level coping strategies, which included sale of assets, borrowing, use of savings, reducing consumption (primarily of food), contracting out farmland, changing jobs, internal migration and intrahousehold labour substitution. They recognised that many of these household-level coping strategies risked weakening their economic security, which exacerbated dilemmas when deciding if, where and when to seek treatment.

### Policy landscape for CL and leprosy

Policy actors credited international organisations and civil society groups’ long-term support of leprosy services in Ethiopia for strengthening the perception that leprosy was treatable through the health service. Key innovations they supported included raising awareness through national mass media campaigns, including World Leprosy Day; involving health extension workers in active case-finding; and, in some hospitals, promoting patient self-care through support groups for affected individuals.

Policy actors felt leprosy care had become more effective with a decentralised approach and that integration of TB and leprosy drugs into regional supply chains had improved treatment services. Nonetheless, they described persistent challenges. They felt early diagnosis remained hampered by over-reliance on clinical diagnosis and lack of consistent availability of laboratory equipment, supplies and training:

There are lots of hotspot [high leprosy endemicity] areas with health centres working there, but unfortunately health centres don’t do the simple [slit] skin smear tests. (KII-72, dermatologist)

Morbidity management resources were previously provided free to affected individuals in South Wollo, but one actor described how their reduced availability had impacted service use:

Shoes, Vaseline, and other things were given to patients, and they came every day. There is no such situation right now. (KII-54, zonal health authority)

Some policy actors felt successful approaches for leprosy could be used for CL, for example, by training health extension workers to build awareness, clinically diagnose CL and refer people to services. In late 2021, integrated NTD prevention and control training sessions for health extension workers throughout Amhara region (including Kalu) covered CL.^4^ However, some policy actors raised concerns about training health extension workers to refer people to health centres because, at a strategic level, health centre staff are not empowered to provide confirmatory diagnosis or treatment for CL. For example, the most recent CL clinical guidance document, which dates from 2013, suggests that slit skin smears, intralesional administration of sodium stibogluconate and liquid nitrogen cryotherapy ‘could’ be delivered by hospitals and health centres, but does not clearly lay out responsibilities at each level of the health system in such a way as to inform implementation strategies for training and equipping staff.^21 22^ Moreover, given the current lack of CL diagnostic and treatment capacity in Kalu, many regional and woreda actors expressed concern about overwhelming health facilities and referral centres if awareness initiatives or active screening exercises resulted in increased treatment-seeking by people with CL:

Decentralizing CL [services] may flood the system unless availability of additional financial and skilled human resources, and supply and diagnostic facilities are put in place. (TB leprosy focal person, speaking at stakeholder meeting in 2022)

At the few dermatology referral centres offering CL care, such as Boru Meda Hospital, policy actors pointed to several challenges for CL treatment that would need to be addressed in future service expansion plans. Challenges included shortages of sodium stibogluconate, a current backlog of cases, high patient costs associated with hospital stays and laboratory investigations and limited staff capacity to follow-up patients over long treatment periods. The effectiveness of CL treatments available for the most common strain of the parasite in Ethiopia, *Leishmania aethiopica*, was also a key concern, which actors at higher levels of the health system linked to underinvestment in clinical research:

This parasite […] is not the same as the parasite found in other countries. It is drug resistant […] That is why, even if we do a lot of advocacy to bring [other drugs] in […] we don't see a significant change in patients. […] larger studies, such as clinical trials, are not conducted in Ethiopia. As a result, we are employing old [CL] treatments from other countries [… or treatments for] visceral [leishmaniasis] […] with a result of around 50% [effectiveness]. (KII-66, government NTD actor, federal level)

Nevertheless, many at our 2022 stakeholder meeting were motivated to address the challenges of how health centres could be used to address CL with available treatments. Health officials considered primary care an important resource to diagnose and treat common skin conditions and that some health centre staff could be supported to perform slit-skin smears to extend CL diagnostic capabilities outside hospitals.^4^ One Amhara regional health official pointed out that some health centres had recently been equipped to deliver carbon dioxide-based cryotherapy for early treatment of cervical cancer; the attendees explored whether this innovation could be repurposed to treat uncomplicated CL.

## Discussion

Our study provides a comprehensive understanding of the local context of disease experiences and care-seeking, health service readiness and policy landscape for both CL and leprosy in Kalu District in Ethiopia. Our data reveal that primary healthcare facilities lacked supplies and equipment, and staff lacked training to diagnose and manage cases of CL, and to a lesser extent leprosy. This absence of provision within primary healthcare has led to low use of facility-based services, but is consistent with policymakers’ expectations, their beliefs around feasibility and their interpretations of national guidelines. Communities held misconceptions both about leprosy and CL transmission; leprosy was thought to be associated with swamp water and CL with bat urine rather than sandflies. These beliefs, however, had little influence on care-seeking choices. People with leprosy commonly sought care from health facilities because long-standing investment in services had delivered effective treatment. In contrast, individuals with CL largely administered self-care or sought care from traditional healers. Their care-seeking choices appeared predominantly driven by awareness of the lack of service availability for CL. Global and national NTD strategies identify CL service expansion and integration as key approaches to address these challenges.^4 10^ Many policy actors felt that supply chain interventions, decentralised treatment approaches and community engagement initiatives for leprosy could also benefit CL, but others also warned against increasing care-seeking from primary healthcare providers and referral centres unless effective and feasible CL treatment could be provided on a scale commensurate with the large burden they perceived.

Another study in Ethiopia has suggested that low public awareness of CL contributes to low care-seeking at facilities.^23 24^ In other contexts, care-seeking delays commonly occur because the early clinical manifestations of CL are misinterpreted.^23 25 26^ These challenges may also be present in some areas of Kalu, but in highland areas, where CL tends to be most endemic, we found that awareness of CL was high and care-seeking was shaped more by people’s awareness that CL services were not available within Kalu—and the financial and opportunity costs to seek care outside the woreda—than by lack of awareness. Our findings echo and reinforce studies describing the costs households face in accessing services for their skin conditions at distant hospitals as a major barrier to care-seeking.^27 28^

To our knowledge, no health facility readiness assessments for leprosy or CL have previously been conducted in Ethiopia, although similarly low levels of primary healthcare facility readiness have been reported previously for other chronic conditions in Ethiopia and elsewhere.^29 30^ We observed health centre readiness to deliver general primary care services to be good, suggesting that lack of service availability was specific to CL and leprosy, rather than simply reflective of a poorly functioning health service. The large differences between health centres and health posts in the availability of basic essential diagnostics, medicines and commodities is an important practical challenge to further decentralisation of services for skin NTDs and community case management.

Our study has several strengths and weaknesses. We were able to combine multiple sources of data across different levels of the health service as well as the views of affected people and of individuals in disease-endemic communities. We integrated a variety of perspectives to understand more fully the impact of these conditions on affected individuals and their communities. Nevertheless, we did not reach every health post in the area, so it is possible that we missed important insights about service provision in these most inaccessible areas. The absence of formal diagnostic testing for CL in the district means we relied on anecdotal reporting and the referral of individuals to Boru Meda Hospital to confirm that the study site is truly endemic for CL. Everyone we spoke to with leprosy had active relationships with health facilities and we could not reach people who no longer sought care. While previous evidence suggests that stigma experiences of CL and leprosy are gendered and often mediated through religious coping,^23 31 32^ we were unable to explore in-depth questions around gender dynamics or how Islam affects disease concepts and the landscape of care in Kalu. We did not address the effects of armed conflict on skin NTD experiences and service provision; however, such themes have been explored elsewhere.^33 34^ Finally, our study was focused in one specific geographical area, but we believe that many of our results may be broadly applicable to other areas of Ethiopia.

To provide timely and accessible diagnosis and treatment for CL and reduce economic barriers to care-seeking for both CL and leprosy, our findings indicate that new models of care are needed. The design of such models will need to meet challenges to the FMoH’s CL service expansion strategy and may incorporate integrated approaches to skin NTDs. Based on policy actors’ agreement that carbon dioxide-based cryotherapy used for cervical cancer might be used to treat CL, we have co-developed a decentralised strategy for staff at health centres to manage uncomplicated CL. The supply of carbon dioxide tends to be more reliable than liquid nitrogen currently used for CL cryotherapy at hospitals. The strategy consists of clinical and diagnostic skills training for CL and skin disease in general and equipping and training staff to conduct slit-skin smears, administer carbon dioxide-based cryotherapy for people with uncomplicated CL and refer severe cases. We are piloting this to explore its safety, efficacy, acceptability, feasibility and cost. Additional interventions will be needed for hospitals. A second intervention we have developed is a series of community dialogues that follows a 4-week curriculum designed to increase knowledge of CL and local services and identify champions in the community who can be CL resource people, help refer people to care and take forward future CL interventions.

Supply-side strategies must be combined with community engagement approaches that build on existing knowledge and recognise common concerns to improve communities’ understandings of leprosy and CL and reduce their stigma. Discussion of cryotherapy as a newly available treatment for CL could build on the logics of popular traditional practices to treat CL wounds in Kalu by cauterising with heat and caustic plant medicines. In West Africa, bringing traditional healers into communities of stakeholders in skin NTD care has been effective in improving service use.^35 36^ Discussion of the role of sandflies rather than bats in transmitting CL will lead to questions about how people can protect themselves from sandfly bites; such conversations will need to be handled with care given the lack of evidence both on effective sandfly control strategies^37^ and on the role of bats in harbouring parasites.^38^ Leprosy and CL stigma interventions in Kalu could target the nuances of how people think about contagion before and after treatment, the need to isolate and abstain from sex for CL treatments to work and the need for people with leprosy to avoid farming to avoid exacerbating their condition. This study shows knowledge, attitudes and practices related to CL and leprosy differ substantially. Interventions seeking to integrate communication strategies for multiple skin NTDs must consider communities’ different information needs for different diseases.

Approaches to improving experiences of skin NTDs, particularly where integrated strategies are proposed, are complex and must be developed and evaluated with a clear understanding of their context. Whereas we originally conceived of developing a complex intervention focused on integrated case-finding and management, our careful formative study has led us to focus on addressing the specific challenges of decentralising diagnosis and treatment for CL and other skin diseases to health centres and building community-level awareness about CL and CL services. We recommend that formative studies be conducted in other contexts to guide development of other skin NTD strategies and build a more comprehensive roadmap for skin NTD control and elimination in Ethiopia.

## supplementary material

10.1136/bmjgh-2024-016650 online supplemental file 1

10.1136/bmjgh-2024-016650 online supplemental file 2

10.1136/bmjgh-2024-016650 online supplemental file 3

10.1136/bmjgh-2024-016650 online supplemental file 4

10.1136/bmjgh-2024-016650 online supplemental file 5

10.1136/bmjgh-2024-016650 online supplemental file 6

## Data Availability

Data are available upon reasonable request.

## References

[1] Engelman D, Fuller LC, Solomon AW (2016). Opportunities for Integrated Control of Neglected Tropical Diseases That Affect the Skin. Trends Parasitol.

[2] WHO (2021). Ending the neglect to attain the sustainable development goals: a road map for neglected tropical diseases 2021–2030. https://www.who.int/publications/i/item/9789240010352.

[3] WHO (2023). Global leprosy (Hansen disease) update, 2022: new paradigm – control to elimination. https://www.who.int/publications/i/item/who-wer9837-409-430.

[4] FMoH (2021). The third national neglected tropical diseases strategic plan 2021-2025. https://espen.afro.who.int/system/files/content/resources/Third%20NTD%20national%20Strategic%20Plan%202021-2025.pdf.

[5] Semahegn A, Manyazewal T, Getachew E (2023). Burden of neglected tropical diseases and access to medicine and diagnostics in Ethiopia: a scoping review. Syst Rev.

[6] Tamiru HF, Mashalla YJ, Mohammed R (2019). Cutaneous leishmaniasis a neglected tropical disease: community knowledge, attitude and practices in an endemic area, Northwest Ethiopia. BMC Infect Dis.

[7] Yotsu RR (2018). Integrated Management of Skin NTDs-Lessons Learned from Existing Practice and Field Research. Trop Med Infect Dis.

[8] Carvalho AG de, Tiwari A, Luz JGG (2021). Leprosy and cutaneous leishmaniasis affecting the same individuals: A retrospective cohort analysis in a hyperendemic area in Brazil. PLoS Negl Trop Dis.

[9] Koffi AP, Yao TAK, Barogui YT (2020). Integrated approach in the control and management of skin neglected tropical diseases in three health districts of Côte d’Ivoire. BMC Public Health.

[10] WHO Ending the neglect to attain the sustainable development goals: a strategic framework for integrated control and management of skin-related neglected tropical diseases. https://www.who.int/publications/i/item/9789240051423.

[11] Sousa P de, Sousa A de, Turchi MD (2021). Reviewing the therapeutic management of leprosy in primary care: demand case series referred to a University Hospital in the Midwest region of Brazil. An Bras Dermatol.

[12] Abeje T, Negera E, Kebede E (2016). Performance of general health workers in leprosy control activities at public health facilities in Amhara and Oromia States, Ethiopia. BMC Health Serv Res.

[13] Phillips RO, Owusu L, Koka E (2024). Development of an integrated and decentralised skin health strategy to improve experiences of skin neglected tropical diseases and other skin conditions in Atwima Mponua District, Ghana. PLOS Glob Public Health.

[14] CSA (2020). Population size by sex, area and density by Region, Zone and Wereda. https://www.statsethiopia.gov.et/wp-content/uploads/2020/08/Population-Projection_Weredas-as-of-July-2020.pdf.

[15] WHO (2021). WEB annex 1: protocol for evaluating minimum package of care of morbidity management and disability prevention for lymphoedema management is designated health facilities | InfoNTD. https://www.infontd.org/resource/web-annex-1-protocol-evaluating-minimum-package-care-morbidity-management-and-disability.

[16] WHO (2015). Service availability and readiness assessment (SARA). https://www.who.int/data/data-collection-tools/service-availability-and-readiness-assessment-(sara).

[17] Tong A, Sainsbury P, Craig J (2007). Consolidated criteria for reporting qualitative research (COREQ): a 32-item checklist for interviews and focus groups. Int J Qual Health Care.

[18] MAXQDA (2022). All-in-one qualitative & mixed methods data analysis tool. https://www.maxqda.com/.

[19] Hailemichael Y, Novignon J, Owusu L (2024). The role of economic factors in shaping and constituting the household burden of neglected tropical diseases of the skin: Qualitative findings from Ghana and Ethiopia. Soc Sci Med.

[20] Haakenstad A, Irvine CMS, Knight M (2022). Measuring the availability of human resources for health and its relationship to universal health coverage for 204 countries and territories from 1990 to 2019: a systematic analysis for the Global Burden of Disease Study 2019. The Lancet.

[21] FMoH (2024). Guideline for treatment & prevention of leishmaniasis in Ethiopia. https://medbox.org/document/guideline-for-treatment-prevention-of-leishmaniasis-in-ethiopia.

[22] van Henten S, Tesfaye AB, Abdela SG (2021). Miltefosine for the treatment of cutaneous leishmaniasis-A pilot study from Ethiopia. PLoS Negl Trop Dis.

[23] Polidano K, Parton L, Agampodi SB (2022). Community Engagement in Cutaneous Leishmaniasis Research in Brazil, Ethiopia, and Sri Lanka: A Decolonial Approach for Global Health. Front Public Health.

[24] Tesfay K, Mardu F, Berhe B (2021). Household knowledge, practice and treatment seeking behaviors towards cutaneous leishmaniasis in the endemic rural communities of Ganta- afeshum district, Tigrai, northern Ethiopia, 2019: a cross-sectional study. Trop Dis Travel Med Vaccines.

[25] McCollum R, Berrian H, Theobald S (2022). Barriers and Enablers to Health-Seeking for People Affected by Severe Stigmatising Skin Diseases (SSSDs): A Scoping Review. Soc Sci (Basel).

[26] Gunasekara SD, Wickramasinghe ND, Agampodi SB (2023). We do not rush to the hospital for ordinary wounds (suḷu tuvāla): A qualitative study on the early clinical manifestations of cutaneous leishmaniasis and associated health behaviours in rural Sri Lanka. PLoS Negl Trop Dis.

[27] Gordon LG, Elliott TM, Wright CY (2016). Modelling the healthcare costs of skin cancer in South Africa. BMC Health Serv Res.

[28] Patcharanarumol W, Siengsounthone L, Vonglokham M (2012). Household costs associated with health care seeking at three tertiary care hospitals in Lao PDR. Southeast Asian J Trop Med Public Health.

[29] Mulugeta TK, Kassa DH (2022). Readiness of the primary health care units and associated factors for the management of hypertension and type II diabetes mellitus in Sidama, Ethiopia. PeerJ.

[30] Asemahagn MA, Alene GD, Yimer SA (2020). Geographic Accessibility, Readiness, and Barriers of Health Facilities to Offer Tuberculosis Services in East Gojjam Zone, Ethiopia: A Convergent Parallel Design. Res Rep Trop Med.

[31] Wenning B, Price H, Nuwangi H (2022). Exploring the cultural effects of gender on perceptions of cutaneous leishmaniasis: a systematic literature review. Glob Health Res Policy.

[32] Beatriz L (2020). Marana: leishmaniasis and the pharmaceuticalization of war in Colombia. https://yorkspace.library.yorku.ca/items/50286c9c-057d-438b-9ffb-da9e0447a1e2.

[33] Ebenso B, Newell J, Emmel N (2019). Changing stigmatisation of leprosy: an exploratory, qualitative life course study in Western Nigeria. BMJ Glob Health.

[34] Arage MW, Kumsa H, Asfaw MS (2023). Exploring the health consequences of armed conflict: the perspective of Northeast Ethiopia, 2022: a qualitative study. BMC Public Health.

[35] Owusu L, Tuwor RD, Ackam N (2023). Role and capacity needs of community based surveillance volunteers in the integrated management of skin neglected tropical diseases (skin NTDs): a qualitative study from central Ghana. BMC Public Health.

[36] Krah E, de Kruijf J, Ragno L (2018). Integrating Traditional Healers into the Health Care System: Challenges and Opportunities in Rural Northern Ghana. J Community Health.

[37] Garlapati R, Iniguez E, Serafim TD (2021). Towards a Sustainable Vector-Control Strategy in the Post Kala-Azar Elimination Era. Front Cell Infect Microbiol.

[38] Austen JM, Barbosa AD (2021). Diversity and Epidemiology of Bat Trypanosomes: A One Health Perspective. Pathogens.

