# Supplementary material for: Understanding experiences of neglected tropical diseases of the skin: a mixed-methods study to inform intervention development in Ethiopia
Source: BMJ Glob Health. 2025 Feb 5;10(2):e016650. doi: 10.1136/bmjgh-2024-016650 (PMC11800212; doi:10.1136/bmjgh-2024-016650)
Supplement: online supplemental file 1 [file bmjgh-10-2-s001.pdf]

Supplementary file 1. Recruitment strategy and socio-demographic profiles of participants for qualitative community-based and policy landscape research

Participant type	Data collection method & number conducted	Data collection method & number analysed	Participant mix in analysed sample	Recruitment strategy
Community leaders	33 interviews	13 interviews	9 men, 4 women	Interviews held with 3-4 leaders (administrative, religious, youth or women's health development army) in each cluster
Community members	40 group discussions	19group discussions	3 women's groups, 7 men's groups, 1 primary school girls' group, 3 primary school boys' groups, 1 female teachers group, 3 male teachers groups, 1 community leaders group	4-5 FGDs were held in each cluster, with discussions for adults/youth and females/males held separately

People with Leprosy	37 interviews	24 interviews	15 men, 8 women, 1 child <18y confirmed to have and treated for leprosy	Potential participants who were confirmed to have leprosy using MoH diagnostic definitions and currently under the care of health facilities for medication or management of their condition were identified from health facility records. This included all 30 people with new infections recorded within Kalu during the previous 12 month data collection period and some diagnosed prior to this and continuing to receive care. Participants were selected purposively based on their consistent presentation to health facilities and geographic accessibility. They were traced by community health extension workers for an interview at their place of residence. All those approached agreed to the interview.
People with CL	33 interviews	18 interviews	11 men, 4 women, 3 children <18y presumed to have CL	Potential participants were identified through conversations with staff at health centres and health posts across clusters, kebele leaders (beginning in lead villages of each cluster), as well as snowball sampling from other people presumed to have CL. Since no CL diagnoses were recorded in health facility records, all potential participants were based on key informants' observations of people in the community and all diagnoses were presumptive (rather than confirmed using

				MoH syndromic or laboratory definitions). Around one-third of people had an active lesion at the time of interview (in the rest, lesions had resolved but scars remained).
Traditional healers	7 interviews	7 interviews	7 men from Ketetya (2), Harbu (1), Gerba (1) and Degan (3), all worked with plant medicines	Healers with some involvement in caring for skin conditions were identified through snowballing, starting by asking community leaders, other research participants and through seeing their services advertised in towns. Several of the healers belonged to a woreda level association for healers and had trained under the same Islamic medical practitioner.
Healthcare workers	23 interviews	12 interviews	7 men, 5 women (community-level TB/Leprosy focal persons, nurses and health officers at heath centres, health extension workers from health posts)	One staff member from all government health facilities participating in readiness assessments

Policy actors	25 interviews	25 interviews	Actors came from national (9), regional (1), zonal (4) and kebele (11) levels including leprosy/TB, CL and NTDs focal persons, government human resources and pharmaceutical supply chain coordinators, health department planning leads, health centre directors (who oversee a health centre and affiliated health posts), and representatives from the WHO and NGOs involved in NTD work	Informants purposefully recruited from among those most involved in organising NTDs service provision at woreda, zonal, regional and national levels
