# Supplementary material for: Understanding experiences of neglected tropical diseases of the skin: a mixed-methods study to inform intervention development in Ethiopia
Source: BMJ Glob Health. 2025 Feb 5;10(2):e016650. doi: 10.1136/bmjgh-2024-016650 (PMC11800212; doi:10.1136/bmjgh-2024-016650)
Supplement: online supplemental file 2 [file bmjgh-10-2-s002.pdf]

Supplementary file 2. Policy documents reviewed

Document	Web link (where available)
Health Policy of the Transitional Government of Ethiopia (1993)	http://repository.iifphc.org/handle/123456789/719
HSDP I - Health Sector Development Program (1997/98- 2001/02): Final evaluation report	Unpublished document
HSDP II - Health Sector Development Program II (2002/ 03 - 2004/ 05): Strategic plan	Unpublished document
HSDP III - Health Sector Development Program III (2005/6-2009/10): Strategic plan	http://repository.iphce.org/xmlui/handle/123456789/2646
HSDP IV - Health Sector Development Program IV (2010/11 – 2014/15): Strategic plan	http://repository.iifphc.org/handle/123456789/218
HSTP I - Health Sector Transformation Plan I (2015/16 - 2019/20)	http://repository.iifphc.org/handle/123456789/285
HSTP II - Health Sector Transformation Plan II (2020/21 - 2024/25)	http://repository.iifphc.org/handle/123456789/1414
NTDs I - National master plan for neglected tropical diseases (2013-2015)	Unpublished document
NTDs II - Second edition of national neglected tropical diseases master plan (2015/16 - 2019/20)	http://repository.iifphc.org/handle/123456789/1333
NTDs III - Third National Neglected Tropical Diseases Strategic Plan (2021-2025)	https://espen.afro.who.int/system/files/content/resources
Ethiopia Sustainability Action Plan for NTD Control, Elimination, and Eradication (2021-2025)	https://espen.afro.who.int/system/files/content/resources

TB & leprosy I - National strategic plan for tuberculosis and leprosy control (2013-2020)	http://repository.iifphc.org/handle/123456789/1410
TB & leprosy II - National strategic plan for tuberculosis and leprosy control (2021-2026)	http://repository.iifphc.org/bitstream/handle/123456789/1548/TBL-NSP-02-September-2020.pdf?sequence=1&isAllowed=y
Guideline for management of TB, DR-TB and leprosy in Ethiopia, 6th edition (2017)	https://www.afro.who.int/sites/default/files/2019-04/Ethiopia-National%20guidelines%20for%20TB%2C%20DR-TB%20and%20Leprosy%20in%20Ethiopia%20-%20Sixth%20Edition.pdf
Guideline for diagnosis, treatment and prevention of leishmaniasis in Ethiopia, 2nd edition (2013)	https://www.afrikadia.org/wp-content/uploads/2018/08/VL_Guidelines_Ethiopia_2013.pdf
Integrated NTDs training manual for health extension workers, Amhara State (2020)	Unpublished document
