# Supplementary material for: Understanding experiences of neglected tropical diseases of the skin: a mixed-methods study to inform intervention development in Ethiopia
Source: BMJ Glob Health. 2025 Feb 5;10(2):e016650. doi: 10.1136/bmjgh-2024-016650 (PMC11800212; doi:10.1136/bmjgh-2024-016650)
Supplement: online supplemental file 3 [file bmjgh-10-2-s003.pdf]

Supplementary file 3. COREQ checklist

Consolidated criteria for reporting qualitative studies (COREQ): 32-item checklist

For manuscript: "Understanding experiences of neglected tropical diseases of the skin: a mixed-methods study to inform intervention development in Ethiopia" by Kaba et al.

Framework from: Tong A, Sainsbury P, Craig J. Consolidated criteria for reporting qualitative research (COREQ): a 32-item checklist for interviews and focus groups. *International Journal for Quality in Health Care*. 2007. Volume 19, Number 6: pp. 349 – 357

**No. Item**	**Guide questions/description**	**Response**
**Domain 1: Research team and reflexivity**		
Personal Characteristics		
1. Interviewer/facilitator	Which author/s conducted the interview or focus group?	Hailemichael Y, Alemu AY, Teklu C, Kebebew G and four trained research assistants with continous support from a senior team from Armauer Hansen Research Institute, Addis Ababa University. Guidance on tool development received from partners at London School of Hygiene and Tropical Medicine
2. Credentials	What were the researcher's credentials? E.g. PhD, MD	Interview team consisted of eight interdisciplinary team members including one post-Doctoral fellow, one PhD student, two master's degree holders, two master's students and two bachelor degree holders

3. Occupation	What was their occupation at the time of the study?	University lecturers, students and researchers
4. Gender	Was the researcher male or female?	Both male and female although the number of females was limited to 2
5. Experience and training	What experience or training did the researcher have?	While senior researchers have established experiences in research and one with advanced qualitative competence and experience, the interview team despite their varied level of education were provided with an extensive training in data collection with application of KIIs, IDIs, FGDs and observation.
Relationship with participants		
6. Relationship established	Was a relationship established prior to study commencement?	Before the lanch of data colleciton key community influential leaders were engaged within the respective communities selected for the research. During the forum, discussions focused on the purpose of the research, expectations from local leaders to facilitate community entry and the community as providers of evidnece as well as implicaions of the study outcome. At health facility level, key management members were orineted about the research, expectaions and implicaions

7. Participant knowledge of the interviewer	What did the participants know about the researcher? e.g. personal goals, reasons for doing the research	General research objectives were clearly explained, as well as the role of the interviewer and note taker. There is no established knowledge of the researchers by the participants
8. Interviewer characteristics	What characteristics were reported about the interviewer/facilitator? e.g. Bias, assumptions, reasons and interests in the research topic	Who the interviewer/facilitator is, where she/he came from, how the interview is conducted were reported
**Domain 2: study design**		
Theoretical framework		
9. Methodological orientation and Theory	What methodological orientation was stated to underpin the study? e.g. grounded theory, discourse analysis, ethnography, phenomenology, content analysis	Concurrent design of a mixed methods study with results triangulated. Qualitative data was analysed mainly using content analysis.
Participant selection		
10. Sampling	How were participants selected? e.g., purposive, convenience, consecutive, snowball	Supplementary File has detailed information about recruitment and sampling.<br><br>Participants for interviews were identified based on their recognized position in the community (authorities and opinion leaders) and health

		facilities (health workers), through health facility records (leprosy-affected people) and snowballing (CL-affected people, traditional healers).<br><br>For FGDs participants were recruited purposively based on their experiences and positions in the community using snowballing and using information from Health Extension Workers who live in the community
11. Method of approach	How were participants approached? e.g. face-to-face, telephone, mail, email	Some policy actors were approached via email, all other participants were approached face-to-face
12. Sample size	How many participants were in the study?	19 FGDs with 6-10 participants per group, 42 interviews with leprosy and CL affected people, 13 community leaders, 12 health workers, 7 traditional healers, 25 policy actors.
13. Non-participation	How many people refused to participate or dropped out? Reasons?	Because of busy schedules, 1 policy actor approached for an interview could not participate
Setting		
14. Setting of data collection	Where was the data collected? e.g. home, clinic, workplace	FGD sessions were conducted in communal space (in school compound or health center compound or open field under a shed),

		while interviews were conducted in the participants home or place of work but in private space.
15. Presence of non-participants	Was anyone else present besides the participants and researchers?	No one present during the FGD sessions and interviews. Caregivers were sometimes present and invited to provide additional information during interviews with children.
16. Description of sample	What are the important characteristics of the sample? e.g. demographic data, date	Both gender (M and F), people with and without formal education and age range of 13 to 90 years participated in interviews at community level. Additionally, NTDs program managers who are involved in skin NTDs decision making or care at district, zonal, regional and national level have participated. The duration of the data collection was from March- -October 2021.
Data collection		
17. Interview guide	Were questions, prompts, guides provided by the authors? Was it pilot tested?	Topic guides for the interviews were developed and further refined during training of research assistant.
18. Repeat interviews	Were repeat interviews carried out? If yes, how many?	One interview with a leprosy-affected person involved multiple interactions; the remainder of interviews were single.

19. Audio/visual recording	Did the research use audio or visual recording to collect the data?	Interviews and FGDs were audio recorded with participant consent and transcribed and translated into English for analysis. One participant did not agree so only field notes were taken.
20. Field notes	Were field notes made during and/or after the interview or focus group?	Scribbles were expanded to field note after the interviews/FGDs while full transcription and translation of voice records started after data collection is completed mid-way of the entire process
21. Duration	What was the duration of the interviews or focus group?	30-90 minutes.
22. Data saturation	Was data saturation discussed?	Yes. The redundancy of information across category of participants were tracked to declare data saturation; for most categories of data collected, only a portion of the data collected was analysed after theoretical saturation was reached.
23. Transcripts returned	Were transcripts returned to participants for comment and/or correction?	No
**Domain 3: analysis and findings**		
Data analysis		
24. Number of data coders	How many data coders coded the data?	Five data coders analysed the data

25. Description of the coding tree	Did authors provide a description of the coding tree?	Yes, code book was developed to provide description of the coding elements
26. Derivation of themes	Were themes identified in advance or derived from the data?	Themes were mainly identified after reading the transcripts while developing the codebook
27. Software	What software, if applicable, was used to manage the data?	MAXQDA plus 2020
28. Participant checking	Did participants provide feedback on the findings?	Key findings were presented to the community representatives, NTDs program managers and CL and leprosy affected people which offered an opportunity to validate the findings
Reporting		
29. Quotations presented	Were participant quotations presented to illustrate the themes/findings? Was each quotation identified? e.g. participant number	Yes with participants ID which remains anonymous
30. Data and findings consistent	Was there consistency between the data presented and the findings?	Yes.

31. Clarity of major themes	Were major themes clearly presented in the findings?	Yes.
32. Clarity of minor themes	Is there a description of diverse cases or discussion of minor themes?	Yes consistent deviations were presented as a contrast to dominant themes/findings
