# Supplementary material for: Understanding experiences of neglected tropical diseases of the skin: a mixed-methods study to inform intervention development in Ethiopia
Source: BMJ Glob Health. 2025 Feb 5;10(2):e016650. doi: 10.1136/bmjgh-2024-016650 (PMC11800212; doi:10.1136/bmjgh-2024-016650)
Supplement: online supplemental file 4 [file bmjgh-10-2-s004.pdf]

Supplementary file 4. Coding framework developed

Diseases	Theme	Sub themes	Explanation (what this encompasses)	Example
Policy landscape for CL and leprosy	Current state of policy landscape on skin health conditions at large and Leprosy – CL in particular<br>Opportunities for integrated response	Availability of policy initiatives towards integrated skin NTD care<br><br>PHC level responses	Opportunities refer to the general and skin NTD (sNTD) specific policies/strategies/programs to integrate care at the national and sub-national level.<br><br>Integrated and/or vertical services targeting sNTDs, and/or CL and leprosy described in stakeholders at national and sub-national level<br><br>Responses at PHC level in terms of availability of diagnostic infrastructure,	Introducing integration as policy agenda, setting CL and leprosy specific targets, and a strategic call for integrated skin NTD strategy<br><br>Current practices of CL and leprosy care:<br><br>Integrated TB and leprosy control program, integrated vector management for leishmaniasis, availability of recording and reporting system

			skilled human resources, supplies and referral pathways	
	Challenges to integrate CL and leprosy care		Challenges or constraints to integrate CL and leprosy care documented in the policy or reported by stakeholder at national and sub-nation level.	Inadequate attention at PHC level to CL control and leprosy elimination targets.<br><br>Poor knowledge of healthcare workers at health center level to diagnose and treat CL
CL	Disease names		The vernacular names the community use to refer to CL	‘Kunchir’ (ቁንጭር) or ‘Konchir’ (ቆንጭር) and ‘Yewef Shint’ (የወፍ ሽንት).
Leprosy			The vernacular names the community use to refer to leprosy	‘Yesiga Dewye’ (የስጋ ደዌ), ‘Kumtna’ (ቁምጥና), ‘Tiliku Beshita’ (ትልቁ በሽታ), ‘Yekoda Beshita’ (የቆዳ በሽታ) and ‘Juzam’ (ጁዛም).
	Disease aetiology			

CL			The causes of disease which the community believed as the cause of CL	Bat body fluids, wrath of God, insect (unspecified) and ‘Shikoko’ (ሽኮኮ) (hyrax), unknown cause
Leprosy			The causes of disease which the community believed as the cause of leprosy	Stagnant and dirty water, heredity, wrath of God, unknown cause, bacteria
CL	Disease transmission		This included how the community describes the transmission of CL, and the community’s belief about the possibility of CL transmission.	Transmittable: Direct contact with bleeding from the lesion, sharing clothes, Not contagious: Only affect the person who have CL without direct transmission from affected person to non-affected person.
Leprosy			This encompassed how the community describes leprosy transmission, and the community’s beliefs	Transmittable before initiation of treatment: Direct contact with

			about the possibility of leprosy transmission.	bleeding, blood from nose or mouth<br>Hereditary transmission<br><br>Not transmittable after initiation of treatment
CL	Signs and symptoms		Signs and symptoms of CL were described as manifestations by the community	Lesion, bleeding, swelling and pain
Leprosy			Signs and symptoms of leprosy were described as manifestations by the community	White discoloration of the skin, cutting foot or hand phalanges
CL	Treatment experiences		Treatment sought by CL affected persons.	Traditional medicine (plants extracts, heat application etc…), home remedies (honey, garlic) and religious treatment (holy soil, tufta….)

Leprosy			Treatment sought by leprosy affected persons	Receive modern treatment from formal health facilities<br>Rarely use traditional treatment
CL	Economic impact of skin NTDs	Financial costs of experiencing illness<br><br>Opportunity costs to individual of experiencing illness<br><br>Opportunity costs to the family of experiencing illness<br><br>Financial costs of seeking care	Impact of experiencing the illness on financial resources of the patient. It does NOT include resources used in care seeking.<br><br>Resources lost by the affected person because of having experienced illness.<br><br>Resources lost by caregivers (family, friends, community members) because of providing care	1.for example, if the patient had to pay to take transport<br>2. For example, it may include lost education, lost earnings, or reduced unpaid labour (e.g. housework, subsistence farming), which the individual could not do either because s/he was physically unable, or because stigma associated with the illness prevented

		Opportunity costs to individual of seeking care<br><br>Opportunity costs to the family of seeking	for the individual experiencing illness.<br><br>Costs associated with the purchase of items related to the treatment of skin condition (eg bandages, dressing, consultation, drugs etc.) This will also include transportation cost to seek treatment.<br><br>Opportunity costs associated with treatment of the skin conditions. These include economic activities that the patient would have to forgo as a result of the condition.	participation in these activities.<br>3. It includes how experiencing the illness has affected education and skills of the caregiver.<br>4.These includes cost of hospital bed, supplies like syringe, gloves and investigations.<br><br>5. For example, if an individual stay away from farming or school to seek treatment). There may also be people who are rendered unemployed because of the skin condition.

			Household members, other relatives or friends who skip work or school to take care of the patient.	6. The opportunity cost of providing this care is the activity forgone.
Leprosy	Economic impact of skin NTDs	Financial costs of experiencing illness<br><br>Opportunity costs to individual of experiencing illness<br><br>Opportunity costs to the family of experiencing Illness	Similar to that of CL	
CL	Coping with the financial and economic burden of skin NTDs	Family/ community-based social support mechanisms<br><br>Borrowing<br><br>Dis-saving	Formal or informal support systems available within the family or community for people with challenges related to an illness. This support may be monetary or non-monetary.	The role of Edir and equb was examined. Likewise, social support mechanisms such as labour contribution which is named “debo or Kire” were used in this case

		Sale of assets<br><br>Reduced consumption	Individual or other members of the household or caregivers have to borrow money from formal or informal sources to cover the cost of this illness?<br><br>Individual or other members of the household or caregivers have to withdraw or take their savings to cover the cost of this illness?<br><br>Individual or other members of the household or caregivers have to sell some assets to cover the cost of this illness?	Borrowing from friends, money lenders and community-based credit organization<br><br>Although not common households used to pay medical bill from what they saved for food consumption.<br><br>Selling life stocks, grain were common coping strategies to overcome the cost of seeking care in this community<br><br>Household food consumption is reduced in

			Individual or other members of the household or caregivers consume less - for example less food or not purchase school books - to cope with the increased economic burden of experiencing or seeking care for skin NTDs?<br><br>Formal insurance	order to pay for care seeking<br><br>Community based insurance has played a role in covering medical expenses although the number of beneficiaries from this scheme is less.
Leprosy	Coping with the financial and economic burden of skin NTDs	In addition to the above strategies leprosy patient used other strategies which CL patients did not implement	Contract out farm land due to physical disability to perform farming activities<br><br>Outmigration to the towns	Leprosy patients were experiencing contracting farm land because of their illness. This make them to earn less income<br><br>Some leprosy patients leave to the town in order to survive by begging

CL	Drivers of stigma	Body appearance<br><br>Suspicion on CL transmissibility	This refers to existence of wounds, scars, deformed body parts due to CL.	Deformed nose or lip, scar on face.<br><br>CL as contagious by sharing utensils
Leprosy		Body appearance<br><br>Fear of contagion	This refers to existence of wounds, scars, deformed body parts due to leprosy.	Lost fingers, deformed nose/ eyes/ arms, dark skin<br><br>Leprosy as contagious by sharing cloths
CL	Forms of stigma	Internalized/self<br><br>Social<br><br>Institutional/structural	Internalized stigma refers to negative feelings and judgments about self-due to anticipated or experienced stigma.<br><br>Social stigma includes acts of labeling and discrimination by other people.	Separating own utensils from families'<br><br>Missing social gatherings.<br><br>Prefers to stay home.<br><br>Denied employment,

			Structural stigma refers to macro-level discriminatory acts underpinned by policies and strategies.	
Leprosy		Internalized/self<br>Social<br>Institutional/structural		Separating own utensils from families'<br><br>Missing social gatherings<br><br>Prefers to stay home.<br><br>Segregated residence for the affected.
CL	Stigmatizers	Family members<br><br>Friends, neighbors<br><br>Strangers	This includes people who practice stigma/ discrimination against people with CL.	Siblings stopping to sleep together.<br><br>Gossips from neighbors,
Leprosy		Family members<br><br>Friends, neighbours	This includes people who practice stigma/	

		Strangers	discrimination against people with leprosy.	
