# Supplementary material for: Understanding experiences of neglected tropical diseases of the skin: a mixed-methods study to inform intervention development in Ethiopia
Source: BMJ Glob Health. 2025 Feb 5;10(2):e016650. doi: 10.1136/bmjgh-2024-016650 (PMC11800212; doi:10.1136/bmjgh-2024-016650)
Supplement: online supplemental file 5 [file bmjgh-10-2-s005.pdf]

Supplementary file 5. Health service readiness for leprosy

Domains, Indicators and scores for Leprosy

Domain	Indicator	Description	Health centre (N=9)	Health post (N=14)	Private clinic (N=20)
A. Training	A1. Trained staff: Diagnosis	At least one clinical health facility staff member trained in diagnosis in the last two years	4	0	2
	A2. Trained staff: Treatment and Management	At least one clinical health facility staff member trained in treatment and management in the last two years	3	0	2
	Average domain score (out of 100) by facility type:		38.9	0	10.0
B. Education	B1. Diagnosis, treatment and management guidelines	At least one guideline covering diagnosis, treatment and/or management reported as available for reference by staff at the health facility	1	0	0
	Average domain score (out of 100) by facility type:		11.1	0	0
C. Diagnostics	C1. Confirmatory diagnostic testing	Diagnosis of leprosy offered at facility (clinical)	9	0	7
	Average domain score (out of 100) by facility type:		100	0	35.0

D. Treatment	D1. Treatment: MDT offered	Leprosy MDT is offered at the facility	8	0	0
	D2. Treatment: MDT in stock	Leprosy MDT is in-stock and viable at the facility	7	0	0
	Average domain score (out of 100) by facility type:		83.3	0	0
F. Patient tracking	F1. Patient tracking system	New suspected and confirmed cases are recorded at facility.	9	2	7
	Average domain score (out of 100) by facility type:		100	14.3	35.0
G. Community management	G1. Active case finding	Health facility undertakes active case finding for leprosy	0	13	0
	G2. Contact tracing	Health facility undertakes contact tracing for leprosy	0	11	0
	Average domain score (out of 100) by facility type:		0	85.7	0
H. Staff knowledge	H1. Staff knowledge: Cardinal signs of PB	Staff member recognises 3 cardinal signs of leprosy	5	0	1
	H2. Staff knowledge: Diagnostic signs of MB	Staff member recognises nodular infiltration, leonine facies and madorosis	0	0	0
	H3. Staff knowledge: Disability identification in leprosy	Staff member recognises sensory loss, anatomical or functional deformities and visual impairment	9	0	0

	H4. Staff knowledge: Treatment	Staff member describes correct treatment regimen and duration for MB and PB leprosy	6	0	0
	Average domain score (out of 100) by facility type:		55.6	0	1.3
Leprosy Readiness Score	Calculated as the average of each domain score (out of 100) by facility type		55.7	14.3	11.6
