# Supplementary material for: Understanding experiences of neglected tropical diseases of the skin: a mixed-methods study to inform intervention development in Ethiopia
Source: BMJ Glob Health. 2025 Feb 5;10(2):e016650. doi: 10.1136/bmjgh-2024-016650 (PMC11800212; doi:10.1136/bmjgh-2024-016650)
Supplement: online supplemental file 6 [file bmjgh-10-2-s006.pdf]

Supplementary file 6. Health service readiness for CL

Domains, Indicators and scores for Cutaneous Leishmaniasis

Domain	Indicator	Description	Health centre (N=9)	Health post (N=14)	Private clinic (N=20)
A. Training	A1. Trained staff: Diagnosis	At least one clinical health facility staff member trained in diagnosis in the last two years	0	0	0
	A2. Trained staff: Treatment and Management	At least one clinical health facility staff member trained in treatment and management in the last two years	0	0	0
	Average domain score (out of 100) by facility type:		0	0	0
C. Diagnostics	C1. Clinical diagnosis	Clinical diagnosis of CL offered at facility	2	0	2
	C2. Confirmatory diagnostic testing	Laboratory case confirmation conducted for all patients (FNA, lesion scraping, skin slit smear)	0	0	0
	C3. Referral	Appropriate onwards referral for treatment or management	3	13	10
	Average domain score (out of 100) by facility type:		18.5	31.0	20.0
D. Treatment	D1. Treatment: CL wound dressing	CL wound dressing, analgesics and antiseptics are available	9	5	0

	D2. Treatment: CL treatments available	Topical treatment, IV antimonials, IV treatments, cryotherapy or intralesional injections in-stock and viable from this facility	0	0	0
	Average domain score (out of 100) by facility type:		50.0	11.9	0
CL Readiness Score	Calculated as the average of each domain score (out of 100) by facility type		22.8	16.1	6.7
